# A Frameshift Mutation within *LAMC2* Is Responsible for Herlitz Type Junctional Epidermolysis Bullosa (HJEB) in Black Headed Mutton Sheep

**DOI:** 10.1371/journal.pone.0018943

**Published:** 2011-05-04

**Authors:** Stefanie Mömke, Andrea Kerkmann, Anne Wöhlke, Miriam Ostmeier, Marion Hewicker-Trautwein, Martin Ganter, James Kijas, for the International Sheep Consortium, Ottmar Distl

**Affiliations:** 1 Institute for Animal Breeding and Genetics, University of Veterinary Medicine, Hannover, Germany; 2 Clinic for Swine and Small Ruminants, Forensic Medicine and Ambulatory Service, University of Veterinary Medicine, Hannover, Germany; 3 Institute for Pathology, University of Veterinary Medicine, Hannover, Germany; 4 Commonwealth Scientific and Industrial Research Organisation Livestock Industries, St. Lucia, Brisbane, Queensland, Australia; The University of Hong Kong, Hong Kong

## Abstract

Junctional epidermolysis bullosa (JEB) is a hereditary mechanobullous skin disease in humans and animals. A Herlitz type JEB was identified in German Black Headed Mutton (BHM) sheep and affected lambs were reproduced in a breeding trial. Affected lambs showed skin and mucous membranes blistering and all affected lambs died within the first weeks of life. The pedigree data were consistent with a monogenic autosomal recessive inheritance. Immunofluorescence showed a reduced expression of laminin 5 protein which consists of 3 subunits encoded by the genes *LAMA3*, *LAMB3* and *LAMC2*. We screened these genes for polymorphisms. Linkage and genome-wide association analyses identified *LAMC2* as the most likely candidate for HJEB. A two base pair deletion within exon 18 of the *LAMC2* gene (FM872310:c.2746delCA) causes a frameshift mutation resulting in a premature stop codon (p.A928*) 13 triplets downstream of this mutation and in addition, introduces an alternative splicing of exon 18 *LAMC2*. This deletion showed a perfect co-segregation with HJEB in all 740 analysed BHM sheep. Identification of the *LAMC2* deletion means an animal model for HJEB is now available to develop therapeutic approaches of relevance to the human form of this disease.

## Introduction

Epidermolysis bullosa (EB) is a heritable heterogeneous group of skin disorders affecting the integrity of the skin and mucosa. Abnormalities of macromolecules which anchor the dermis to the epidermis lead to diminished cohesion of the skin layers, blister formation, and fragility. The condition is triggered by frictional movement as well as minor trauma [1]. This mechanobullous disease is well known in several livestock species and in human. Based on the cleavage levels of skin, EB has been classified into the main groups EB simplex, dystrophic EB and junctional EB (JEB). JEB involves cleavage within the lamina lucida which is a component of the basement membrane [2]. Herlitz type JEB is a lethal condition, whereas non-Herlitz type JEB is rarely related to death. In human, three genes are known to be associated with HJEB including *laminin β3* (*LAMB3*), *laminin γ2* (*LAMC2*) and *laminin α3* (*LAMA3*). The defect is usually inherited in an autosomal recessive manner and also cases of compound heterozygosity were described [3]. In human, nonsense, splicing and deletion mutations of *LAMB3*, *LAMC2* and *LAMA3* have been reported as responsible for HJEB [4]. In domesticated animals, cases of JEB are described in horses [5]–[7], dogs [8]–[11] and cats [12]. In German Pointer dogs, a homozygous insertion of repetitive satellite DNA within intron 35 of *LAMA3* was shown to be causal for the defect [10]. Another study in a French subpopulation of this dog breed gave evidence for a non-conservative change of 14 amino acids within *LAMA3* causing JEB [11]. In Belgian draft horses, Comtois horses, and Breton horses affected by HJEB, a homozygous insertion of one nucleotide within exon 10 of *LAMC2* was detected [5], [6]. Furthermore, in American Saddlebred horses HJEB was reported to be caused by a homozygous deletion spanning exons 24–27 of *LAMA3* [7]. In sheep, cases of dystrophic EB have been described in the breeds Weisses Alpenschaf and Assaf sheep [13], [14]. In German Black Headed Mutton (BHM) sheep ovine HJEB has been ascertained in several flocks and after performing a breeding trial with a sample of parents which gave birth to HJEB-affected lambs, a monogenic autosomal recessive inheritance was most likely [15].

In this study, we employed animals from farms and a breeding trial to identify the mutation causing ovine HJEB in German BHM sheep. The most likely candidate gene *LAMC2* was identified using the Illumina OvineSNP50 Beadchip and candidate gene-associated polymorphisms. We provide the complete ovine coding sequence of *LAMC2* and characterize the transcriptional effects of the *LAMC2*-mutation responsible for HJEB.

## Results

### Phenotype

**Figure 1:**
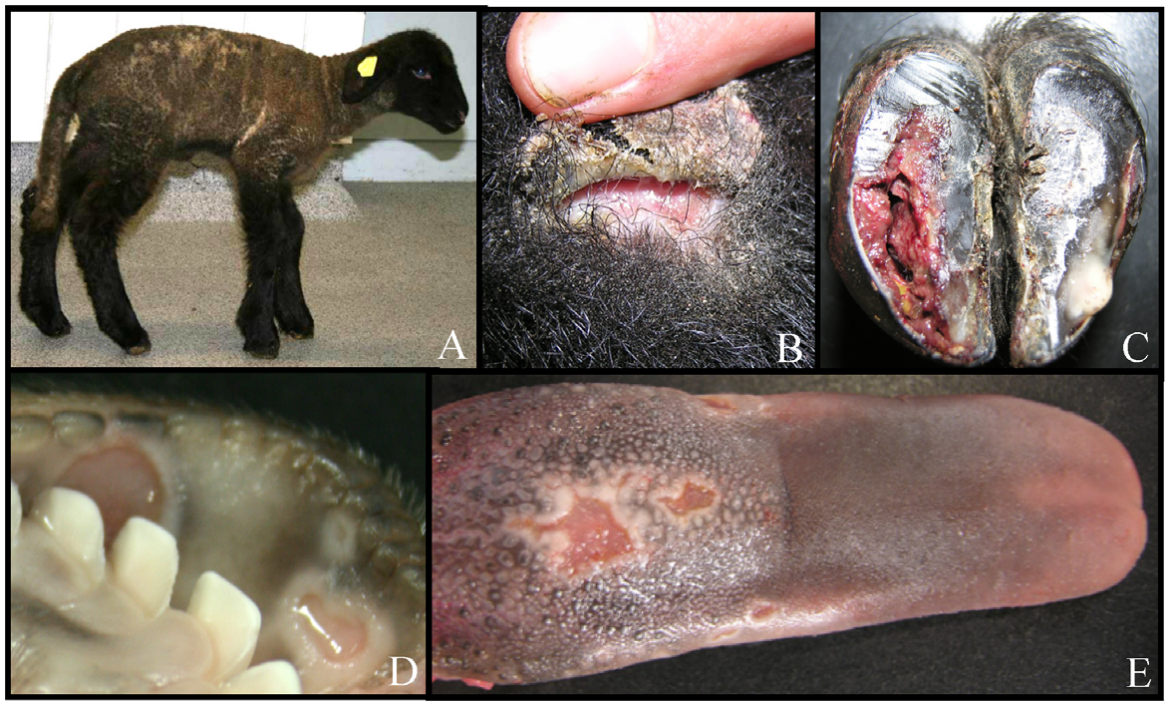
Clinical and pathomorphological feature of HJEB in German Black Headed Mutton sheep. Typical signs are a kyphotic attitude and severe lameness (A), detachment of the epidermis from the underlying dermis (B, dorsal carpal joint), detachment of hoof horn from the corium and accumulation of bloody fluid in the cavity (C), erosion of mucous membranes (D) and of the tongue (E).

The clinical examination of 21 HJEB affected lambs revealed typical signs as shedding of hoof horn, erosion and ulcers of the skin as well as the mucous membranes (Figure 1). All affected lambs had to be euthanised due to the progressive deterioration of their condition. Electron microscopy showed that the separations of the dermoepidermal junction were located in the lamina lucida of the basement membrane. Hemidesmosomes were present in reduced numbers. Immunofluorescence showed a markedly reduced expression of laminin 5 protein whilst expression of collagen VII appeared to be normal. Laminin 5 consists of three subunits encoded by the genes *LAMA3*, *LAMB2* and *LAMC2* [4], which consequently were chosen for scanning polymorphisms that could be used for linkage and association analyses.

### Mapping the Causative Gene

The ovine genome assembly v2.0 (http://www.livestockgenomics.csiro.au/) located *LAMA3* on ovine chromosome (OAR) 23 at 34.8 Mb, *LAMB3* on OAR12 at 78.8 Mb and *LAMC2* also on OAR12 at 68.6 Mb. These genes were partly sequenced to detect polymorphisms, which were subsequently used for linkage analyses in the 21 affected sheep and their direct relatives. Within *LAMA3* (FM872294), seven single nucleotide polymorphisms (SNPs) and one microsatellite were detected (Table S1). Of these markers, five were in a common linkage group and the remaining two in another one. None of these markers showed linkage with HJEB in the data analysed here. Within *LAMB3* (FM872309), 15 SNPs distributed on seven linkage-disequilibrium blocks (Table S1) and within *LAMC2* (FM872310), five SNPs within four linkage-disequilibrium blocks were detected and analysed (Table 1). The chromosome-wide p-values for linkage with HJEB were at *p* = 0.004 for *LAMB3*-associated SNPs and for *LAMC2*-associated SNPs at *p*<0.00001. Strong linkage disequilibrium among the markers within both genes obviated a further resolution among the two candidate genes. Thus, all these gene-associated SNPs were tested for association with HJEB. None of the markers within *LAMA3* and *LAMB3* showed a significant association with HJEB, while three of the five SNPs within *LAMC2* were significantly associated with HJEB (*p* = 0.0003). In addition to the analysis of candidate gene-associated markers, we performed a genome-wide association study to validate linkage to *LAMC2* and determine if additional genomic regions displayed evidence of association. DNA from 12 HJEB-affected BHM lambs (cases), 6 HJEB-unaffected BHM sheep representing parents or full-sibs of the cases and 6 control samples from the whole BHM population were genotyped using the *ovine* Illumina 50 K beadchip. Quality control for genotyping was performed using two duplicates. Consistency of genotyping was >0.999. After filtering for a minor allele frequency >0.05 and genotyping rate >0.99, 43,130 SNPs (single nucleotide polymorphisms) were left for a genome-wide association analysis among cases and controls. After genome-wide permutation to correct for multiple testing, only 11 SNPs were genome-wide significant (*p*<0.05). All these hits were located on ovine chromosome (OAR) 12 in a region spanning 63.1–70.4 Mb which contains the *LAMC2* gene (at 68.8 Mb). The strongest association (*p* = 0.0001) with HJEB was obtained for SNP *OAR12_65207540, OAR12_65811822, OAR12_66169798 and OAR12_s45876*. A mixed linear model analysis including the fixed effect of the sex of the animal and the random animal effect parameterized through the identical-by-state relationship matrix gave the highest hits at 67.333 Mb (−log_10_ p-value = 22.4) and at 68.951 Mb on OAR12 (−log_10_ p-value = 21.1) as shown in the Q-Q and Manhattan plots of the −log_10_ p-values (Figure S1). Therefore, *LAMC2* was confirmed through analysis of gene-associated SNPs and genome-wide SNPs as the most likely candidate harbouring the causal mutation for HJEB.

**Table 1:** SNP motifs for the SNPs detected within *LAMC2*, their surrounding sequence, their effect on the protein sequence and their p-values of association with Herlitz type junctional epidermolysis bullosa (HJEB) in 73 Black Headed Mutton (BHM) sheep.

Polymorphism	Location within*LAMC2*	Wildtype sequence	Mutated sequence	Amino acid exchange	P-value(−log_10_)
FM872310:c.367C>T	exon 3	TTCCATACC**C**TCACCGAT	TTCCATACC**T**TCACCGAT	p.L123F	7.6
FM872310:c.570C>T	exon 5	GTTTTTGCTA**C**GGGCATT	GTTTTTGCTA**T**GGGCATT	-	
FM872310: c.1523G>A	exon 11	GGGGAAC**G**TGGTCCAGT	GGGGAAC**A**TGGTCCAGT	p.R508H	3.3
FM872310: c.1665T>C	exon 11	GTGCAAAGC**T**GGCTACT	GTGCAAAGC**C**GGCTACT	-	
FM872310: c.1878A>G	exon 13	GTTTATGCA**A**CAACTTGA	GTTTATGCA**G**CAACTTGA	-	
FM872310: c.1893T>C	exon 13	GAGAGCCTTGAGACACT	GAGAGCCT**C**GAGACACT	-	
FM872310: c.1923T>C	exon 13	CTCAGGCTGG**T**GGAGGA	CTCAGGCTGG**C**GGAGGA	-	
FM872310: c.2358T>C	exon 16	TGAAGACTA**T**TCCAAGCA	TGAAGACTA**C**TCCAAGCA	-	
FM872310: c.2546A>G	exon 17	ATAGTCTCC**A**CCTTCTCA	ATAGTCTCC**G**CCTTCTCA	p.H849R	0.4
FM872310: c.2746delCA	exon 18	AATGAGAGA**C**AGAAATCA	AATGAGAGA::GAGAAATCA	p.Q916EfsX13	14.8
FM872310: c.3000T>C	exon 20	TGCTGCTAC**T**GACGCCCA	TGCTGCTAC**CG**ACGCCCA	-	
FM872310: c.3001G>A	exon 20	GCTGCTACT**G**ACGCCCA	GCTGCTACT**A**ACGCCCA	p.D1001N	10.1
FM872310: c.3258T>C	exon 22	AGCAGAGC**T**GAGAATGC	AGCAGAGC**C**GAGAATGC	-	
FM872310: c.3455G>A	exon 23	GCAGTGGG**G**CCACCTCC	GCAGTGGG**A**CCACCTCC	p.G1152D	9.9
FM872310: c.3483C>A	exon 23	ACAAGCAT**C**GATGGCATT	ACAAGCAT**A**GATGGCATT	-	
FM872310: c.3600T>C	3′UTR	TTTCCCAAC**T**GGGGTTCTT	TTTCCCAAC**C**GGGGTTCTT	-	
FM872310: c.2749+51A>G	intron 18	ATGGCTACC**A**GTAAGAAGA	ATGGCTACC**G**GTAAGAAGA	*	12.9

*Alternative splicing (only in combination with c.2746delCA deletion): p.Q916EfsX14.

The c.2746delCA mutation causes HJEB due to a premature stop codon.

### Mutation Analysis

After transcription of mRNA into cDNA, the complete coding sequence of *LAMC2* was obtained in two unaffected and two HJEB-affected sheep. We identified a 3,576 bp open reading frame (ORF) in the healthy sheep, which corresponds to the bovine ORF of *LAMC2*. The ovine ORF sequence of the unaffected sheep had an identity of 96.8% with the orthologous bovine sequence. Comparing the sequences of unaffected and HJEB-affected sheep, we detected 14 SNPs and a 2-bp deletion. Of these polymorphisms, six were non-synonymous (c.367C>T, c.1523G>A, c.2546A>G, c.2746delCA, c.3001G>A, c.3455G>A) and led to an alternate amino acid sequence (p.L123F, p.R508H, p.H849R, p.Q916EfsX13, p.D1001N, p.G1152D) (Table 1). All non-conservative polymorphisms were analysed in all 21 HJEB-affected lambs and their relatives. The polymorphisms c.3001G>A and c.3455G>A were in the same linkage phase in all tested animals, while the other SNP alleles showed lower correlations with r^2^<0.8 (Figure S2). Only the c.2746delCA mutation showed a perfect co-segregation with HJEB and allows detection of all affected individuals, unaffected wildtype individuals and heterozygous carriers of HJEB in BHM sheep (Table 2). The χ^2^-values for genotypic and allelic distribution were at 68.0 and 50.4, respectively, and the corresponding p-values were at *p*<0.00001.

**Table 2:** Distribution of the *LAMC2* c.2746delCA mutation in Black Headed Mutton (BHM) sheep from the breeding experiment, random samples of HJEB-unaffected BHM, Suffolk, East Friesian and Leine sheep.

c.2746delCA genotype	BHM (breeding trial)		BHM	Suffolk	East Friesian	Leine
	HJEB-affected (n = 21)	Relatives of HJEB-affected (n = 52)	Random sample (n = 645)	Random sample (n = 175)	Random sample (n = 100)	Random sample (n = 62)
CA/CA		17	639	175	100	52
del/CA		35	6			
del/del	21					

The c.2746delCA (p.Q916EfsX13) mutation causes a premature stop codon at position p.A928X and an alternative splicing of exon 18 (Figure S3). This deletion is located two base pairs upstream of the boundary between exon 18 and exon 19. The alternative splicing lead to an insertion of the first part of intron 18 into the alternatively spliced coding sequence of *LAMC2* and could be found in all affected animals and carriers. Overlapping cDNA sequences in HJEB-affected sheep were amplified using primer pairs from exon 17 to intron 18 and primer pairs from intron 18 to exon 20. Sequencing of these amplicons in both directions gave evidence of a 51-bp insertion from intron 18 into the alternatively spliced coding sequence and a SNP within intron 18 (c.2749+51A>G). This SNP activates a cryptic splice donor site and terminates the transcription of intron 18 (Figure S4). In order to proof the distribution of the c.2749+51A>G SNP, we genotyped all affected sheep and their relatives for this mutation. All HJEB-affected sheep were homozygous G/G, the heterozygous carriers were also heterozygous at c.2749+51A>G, except for three individuals which were homozygous G/G. The unaffected wildtype sheep were homozygous A/A at c.2749+51A>G, except for four individuals which were heterozygous. Of one of these four wildtype individuals, tissue samples were available for cDNA analysis. These samples were used for PCR with primer pairs spanning from exon 17 to intron 18, from intron 18 to exon 20, and from exon 17 to exon 20. While the amplicon including exon 17 to exon 20 was clearly visible on an agarose gel, the other PCR-products could not be amplified indicating an absence of the alternative splicing. Therefore, the c.2746delCA mutation seems to be necessary for the alternative splicing and the c.2749+51A>G mutation for the size of the alternatively spliced product. The alternatively spliced product contains a stop codon at c.2749+41 (Figure S5).

To estimate the distribution of the mutant allele in the BHM sheep population, 645 randomly sampled, unaffected individuals of this breed were genotyped for the c.2746delCA. An association analysis using all 717 BHM sheep genotyped in this study resulted in χ^2^ values for genotypic and allelic distributions of 713 and 711, respectively. The corresponding p-values were *p* = 1.49e^−155^ and *p* = 1.08e^−156^. Of these sheep genotyped, no animal showed the homozygous mutant genotype and six individuals out of different flocks were heterozygous carriers of HJEB. These results allow the conclusion that about 0.9% of the BHM population are carriers for HJEB.

Since Suffolk sheep have contributed to the foundation of the BHM breed, the deletion identified in BHM sheep was genotyped in 175 unaffected and randomly sampled Suffolk sheep. However, not any of the genotyped animals carried the c.2746delCA mutation. Furthermore, random samples of each 50 white and brown colour variants of the breed East Friesian and 62 Leine sheep were genotyped (Table 2). None of these animals showed the c.2746delCA mutation either.

## Discussion

A 2-bp deletion within exon 18 of *LAMC2* (c.2746delCA) was identified as responsible for HJEB in BHM sheep. This c.2746delCA mutation causes a frameshift with a premature stop codon and an alternative splicing of exon 18. In all sheep exhibiting the c.2746delCA mutation, a second intronic c.2749+51A>G mutation could be shown. As the alternatively spliced RNA did also contain a stop codon 42 bp downstream of c.2746delCA, all HJEB-affected sheep in the present study will have a truncated *LAMC2* protein. The presence of c.2746delCA mutation is sufficient to introduce the alternative splicing process because for individuals alone with the c.2749+51A>G mutation alternatively spliced cDNA could not be shown. We assume that the deletion of CA changes the 5′ splice donor site from GACAG/GT to GAG/GT and due to the missing C, the splice site at the 5′ end of exon 18 is not recognized in each case [16]. The c.2749+51A>G SNP located in intron 18 creates a cryptic splice site by converting the sequence CCAGTAAGA to CCGGTAAGA. Even if this splice site seems not to be perfect compared to the reported splice donor consensus sequences [16], the intronic insert was always terminated at this site. According to the splice site competition model, alternative splicing happens when one donor is sufficiently good to compete with the other donor for U1 snRNA binding [17]. In the case that both mutations are present, the splice sites are competitively used, which results in two different transcripts in sheep possessing these mutations.

*LAMC2* encodes for laminin γ2, which is one of three distinct polypeptides that compose laminin 5. These laminin alpha, beta and gamma polypeptides generate a cruciform structure of three short arms formed by different chains and one long arm composed of all three chains. The long arm of laminin γ2, together with those of laminin α3 and laminin β3, spans the lamina lucida, ending right underneath the basal keratinocytes layer [18]. The short arm region contains the nidogen and fibulin binding sites, which integrate the laminin 5 protein into the basement membrane [19]. Mutations within laminin γ2 are most frequently located within the N-terminal LE domains and the L4 module, whereas mutations in the C-terminal α-helical domain are rare [4]. Compared with the human structure, the sheep mutation c.2746delCA (p.Q916EfsX13) is located within the α-helical domain and shortens the polypeptide chain by a premature stop codon 13 triplets downstream of the deletion. Therefore, this SNP mutation presumably influences the assembling of laminin γ2 with its partner chains leading to a non- or dysfunction of laminin 5.

The BHM breed has been introgressed by Suffolk sheep, which were reported to have shown signs of HJEB [20]. In order to confirm if the mutation was introduced by Suffolk sheep into the BHM breed, Suffolk from different flocks were randomly sampled and genotyped. However, none of the genotyped animals carried the HJEB mutation. This implies that either HJEB was not introduced into the BHM population by Suffolk sheep or the defect is more infrequent in the latter breed or maybe even eradicated. On the other hand, BHM sheep have been crossed into other breeds as white and brown colour variants of the breed East Friesian sheep and the Leine sheep. Therefore we checked random samples of these breeds for the presence of c.2746delCA. All individuals of these breeds were homozygous for the wild type sequence. This might indicate that HJEB has not been transmitted into these breeds or that the allele frequency is very low and the c.2746delCA mutation is limited to BHM. On the other hand, it should be noted that no symptomatic animals from these other breeds have been tested, which leaves open the possibility the c.2746delCA mutation is not confined to BHM or even more likely that other not yet known mutations within *LAMC2, LAMA3 or LAMB3* are causing HJED in other sheep breeds. Though HJEB is known in many species, and causal mutations within *LAMC2* have been identified in the human and horse [4], [6], the c.2746delCA mutation was not reported before and can therefore be regarded as new in this regard (Table S2).

In conclusion, we identified the causal mutation for ovine HJEB in BHM sheep. This mutation (c.2746delCA) is located within *LAMC2* and leads to a frame shift of the ORF and a premature stop codon (p.Q916EfsX13) as well as induces an alternative splicing process for exon 18. The genetic test can be employed for eradication of this lethal mutation in the BHM sheep breed. Furthermore, this study provides a suitable animal model for therapeutic approaches of HJEB caused by a specific mutation. Due to the genetic complexity of HJEB, several animal models seem necessary to work out the specific effects caused by the different mutations within *LAMC2, LAMA3 or LAMB3* and the sheep model proposed allows therapeutic interventions for several weeks before HJEB becomes lethal and in particular, to study the factors causing lethality.

## Materials and Methods

### Ethics Statement

All animal work has been conducted according to the national and international guidelines for animal welfare. The breeding experiment at the University of Veterinary Medicine Hannover was under the supervision of the Lower Saxony state veterinary office Niedersächsisches Landesamt für Verbraucherschutz und Lebensmittelsicherheit, Oldenburg, Germany (registration number 33.42502-05/1023).

### Animals

We obtained blood samples of a total of 21 HJEB affected BHM sheep, of which 19 were purebred and two were crossbred animals with German White Headed Mutton or Leine sheep relatives in second to fourth degree. The diagnosis of HJEB was ascertained by clinical, pathological and histological methods. In addition, immunofluorescence and electron microscopy were employed to distinguish HJEB from the non-Herlitz type of JEB. Fifteen of the HJEB affected lambs were born in three different flocks in Lower Saxony, Germany, and eight ewes from two farms and one ram which had already produced HJEB affected offspring were employed for a breeding experiment at the University of Veterinary Medicine Hannover and under the supervision of the state veterinary office (registration number 33.42502-05/1023). In this breeding experiment six HJEB affected lambs were born. Also 51 samples of unaffected sheep which were closely related with HJEB affected lambs were taken. Genome-wide association analyses were performed using DNA from 12 HJEB-affected BHM lambs (cases), 6 HJEB-unaffected BHM sheep representing parents and full-sibs of the cases and 6 control samples from the whole BHM population. We could not trace back the affected BHM lambs to a common founder due to missing information on some ancestors of the HJEB affected sheep (Figure S6). Furthermore, 645 samples of non-affected BHM sheep from 50 farms as well as 337 samples of non-affected sheep of other breeds as Suffolk sheep (175), East Friesian sheep (100), and Leine sheep (62) were used for genotyping. DNA was extracted from blood and tissue samples using the QIAamp 96 DNA Blood kit (Qiagen, Hilden, Germany). We used skin and lung tissue of each two unaffected, carrier and affected sheep for extraction of RNA. The samples were harvested immediately after euthanasia and asserved in RNAlater RNA Stabilization Reagent (Qiagen) for stabilization and protection of cellular RNA *in situ* and then stored at −20°C. The RNA was extracted from tissue samples using the Nucleospin RNAII-kit (Macherey-Nagel, Düren, Germany) and transcribed into cDNA using SuperScript III Reverse Transcriptase (Invitrogen, Karlsruhe, Germany).

### Sequence and mutation analysis of the ovine *LAMA3*, *LAMB3* and *LAMC2* genes

For analysis of *LAMA3*, *LAMB3* and *LAMC2*, we designed primers with Primer3 software (http://frodo.wi.mit.edu/cgi-bin/primer3/primer3_www.cgi) using sequences derived from the ovine genome assembly v2.0 (http://www.livestockgenomics.csiro.au/) or, if ovine sequences were not available in the targeted region, we designed heterologous primers based on bovine sequences (Table S3). PCR products were sequenced on a MegaBACE 1000 capillary sequencer (GE Healthcare, Freiburg, Germany) and analysed using Sequencher 4.7 (GeneCodes, Ann Arbor, MI, USA). For the *LAMC2* mutation analysis we sequenced overlapping cDNA fragments spanning the entire open reading frame in each two affected and non-affected sheep. All detected polymorphisms were genotyped in the 21 samples of HJEB affected sheep and 51 samples of closely related unaffected sheep. After this, single- and multipoint non-parametric linkage analysis were performed using the Merlin software, version 1.1.2 [21]. The only polymorphism in complete concordance with HJEB in the genotyped animals was sequenced on cDNA and genomic DNA in each two affected, unaffected and carrier animals. Genotyping of c.2746delCA was performed by sequencing as well as by digestion of PCR products using the MnlI enzyme (NEB, Frankfurt, Germany).

### Non-parametric linkage and association analyses

Non-parametric single- and multipoint linkage (NPL) analyses were employed for the HJEB-affected lambs and their closely relatives, which were from three different farms. Sheep from farm I and farm II were connected by common progeny born in the breeding trial (Figure S4) Linkage analysis was based on allele sharing by identical-by-descent methods and the MERLIN 1.1.2 software [21]. The NPL statistics Zmeans and the LOD (logarithm of the odds) scores were employed for detection of allele sharing among affected family members. The minimum (min) and maximum (max) achievable Zmeans and LOD scores were high enough to reach genome-wide significant linkage. For the genome-wide type I error probability (P_g_), a Bonferroni correction was applied with P_genome-wide_ = 1−(1−P_chromosome-wide_)^1/r^, where r = length of OAR12 (86.14 Mb) divided by the total ovine genome length in Mb (2647 Mb). Using this formula, the threshold for P_g_ of 0.05 and 0.01 is at a chromosome-wide p-value of 0.001668 and 0.000327. A significant co-segregation of a marker allele with the phenotypic expression of HJEB in the examined population was assumed for p-values <0.05.

Association of SNPs with HJEB was tested using the CASECONTROL procedure of SAS/Genetics (Statistical Analysis System, version 9.2, Cary, NC, USA, 2010) and PLINK, version 1.07 [22]. Genome-wide significance was obtained using 1,000,000 permutations with the max(T) permutation procedure of PLINK.

Statistical calculation of pairwise linkage disequilibrium (LD) was performed and pictured using HAPLOVIEW 4.0 [23]. We used the Tagger algorithm r^2^≥0.8 to detect SNPs with strong LD among alleles.

A mixed linear model (MLM) was employed to control for sex of the animals and the identity-by-state (IBS) kinship matrix among all individuals (K-matrix) using TASSEL [24]. The KIN option of TASSEL was used to create the K-matrix containing the IBS coefficients. Equations were solved via restricted maximum likelihood.

## Supporting Information

Figure S1 **Results of the genome-wide association analysis.** (A) Q-Q plot of the −log_10_ p-values from a mixed linear model analysis for association of genome-wide SNPs with Herlitz type junctional epidermolysis bullosa (HJEB) in Black Headed Mutton (BHM) sheep. (B) Manhattan-plot of the −log_10_ p-values for the genome-wide association analysis of Herlitz type junctional epidermolysis bullosa (HJEB) in Black Headed Mutton (BHM) sheep. The peaks of the −log_10_ p-values are only on OAR12.
(DOC)

Figure S2 **Linkage disequilibrium (LD) of six non-conservative SNPs within** ***LAMC2*** **.** The r^2^ values are shown for each SNP pair. The red square between the markers c.3001G>A and c.3455G>A indicates complete linkage.
(DOC)

Figure S3 **cDNA sequence of an HJEB-unaffected lamb (A) in comparison with the cDNA sequence of an HJEB-affected lamb (B, C).** The cDNA sequence of an HJEB-affected lamb is spanning from exon 17 to exon 19 (B) and the cDNA sequence of the same affected lamb is spanning from exon 17 to intron 18 (C). The vertical line marks the end of exon 18. In the affected individual (B/C), at the end of exon 18 two bases (CA) are missing and alternative splicing results in two transcripts with one of them including 51 bp of intron 18 (B). The presence of intronic DNA within the second transcript can be clearly shown by generating sequences starting from exon 17 and ending in intron 18 (C). The boundary between exon 17 and exon 18 of all products was smooth and used to verify that only cDNA was sequenced.
(DOC)

Figure S4 **Cryptic intronic splice site in an HJEB-affected lamb.** Section of the cDNA sequence of an HJEB-affected lamb showing the sequence of intron 18, which is spliced at the intronic base pair 51 to exon 19. The A>G mutation opening a cryptic splice site and therefore terminating this intronic insertion into the cDNA sequence is indicated.
(DOC)

Figure S5 **Comparison of amino acid sequences among an unaffected and an HJEB-affected animal.** Sequence labelled with 1 is from an unaffected lamb and sequences labelled 2 and 3 are from one HJEB-affected lamb. Sequence 2 demonstrates the transcript with correct and sequence 3 demonstrates the transcript with alternative splicing. Both variants (2 and 3) were present in every HJEB-affected lamb and both sequences lead to a premature stop codon and thus to a shortened, non-functional protein.
(DOC)

Figure S6 **Pedigree of the HJEB-affected Black Headed Mutton sheep.** Sheep from the three farms are marked by grey boxes in contrast to the progeny born in the mating experiment. Animals marked by an asterisk were genotyped on the ovine Illumina 50 K beadchip.
(DOC)

Table S1 **Polymorphisms identified within ovine** ***LAMA3*** **and** ***LAMB3*** **used for linkage analysis and their location, polymorphism information content (PIC) and heterozygosity (HET).**
(DOC)

Table S2 **Mutations within** ***LAMC2*** **causing Herlitz and Non-Herlitz JEB in human and horse.**
(DOC)

Table S3 **Primer sequences with their product sizes (P) and annealing temperatures (AT) used for amplification of sequences within** ***LAMA3*** **,** ***LAMB3*** **, and** ***LAMC2*** **as well as the targeted regions within these genes.**
(DOC)
