# Supplementary material for: A Frameshift Mutation within LAMC2 Is Responsible for Herlitz Type Junctional Epidermolysis Bullosa (HJEB) in Black Headed Mutton Sheep
Source: PLoS One. 2011 May 4;6(5):e18943. doi: 10.1371/journal.pone.0018943 (PMC3087721; doi:10.1371/journal.pone.0018943)

**Figure S1.** Results of the genome-wide association analysis. (A) Q-Q plot of the –log_10_ p-values from a mixed linear model analysis for association of genome-wide SNPs with Herlitz type junctional epidermolysis bullosa (HJEB) in Black Headed Mutton (BHM) sheep.

(B) Manhattan-plot of the –log_10_ p-values for the genome-wide association analysis of Herlitz type junctional epidermolysis bullosa (HJEB) in Black Headed Mutton (BHM) sheep. The peaks of the –log_10_ p-values are only on OAR12.

**(A)**


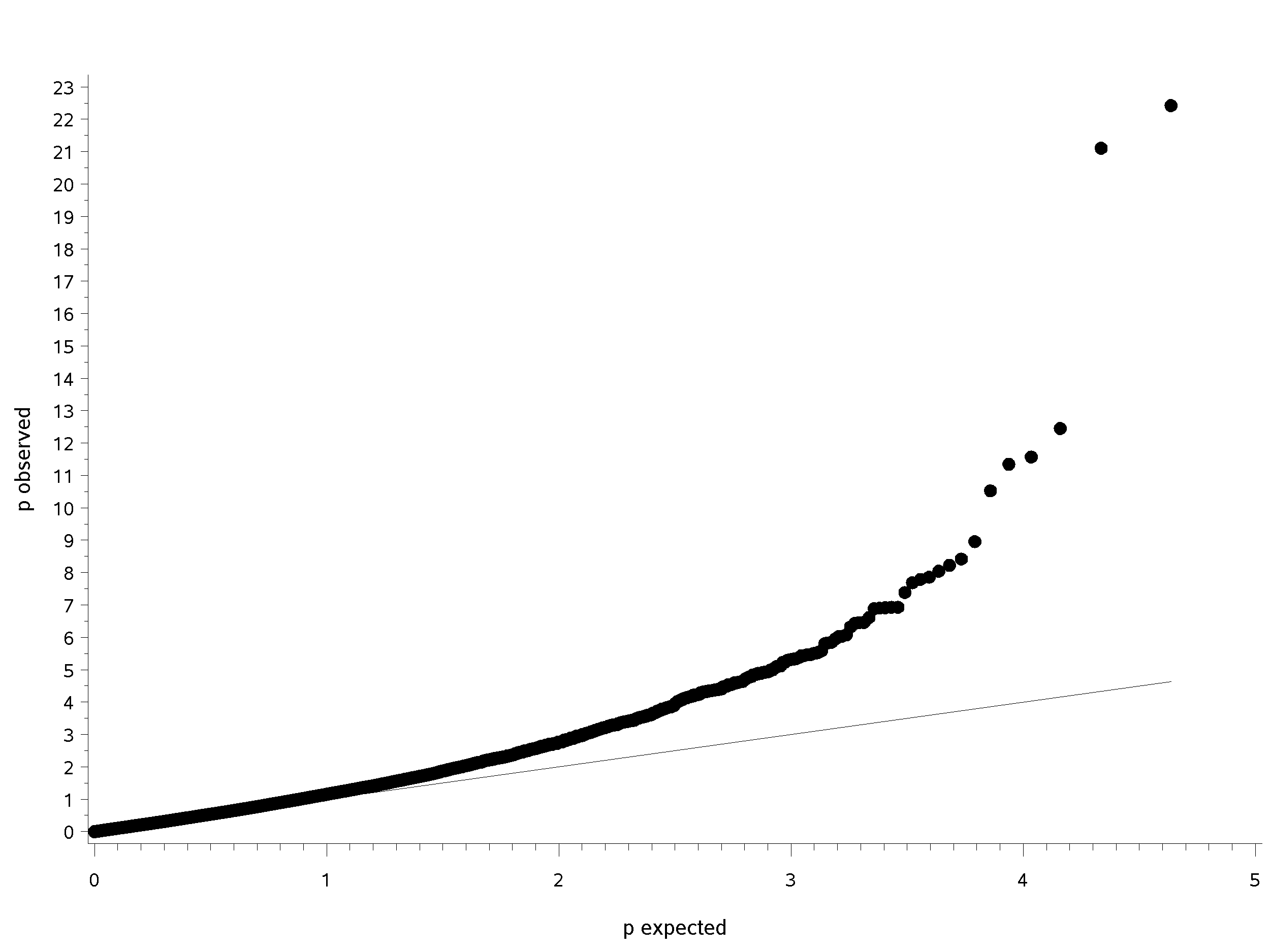


**(B)**


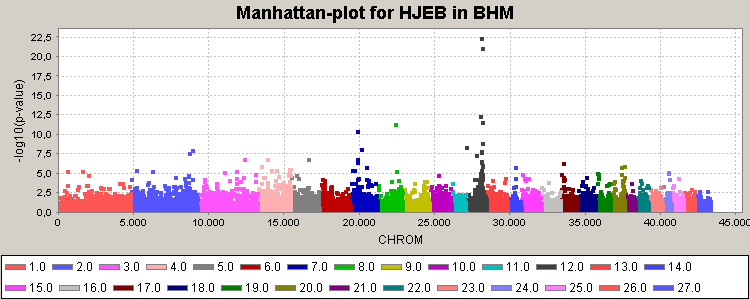

Supplement: Figure S1 — Results of the genome-wide association analysis. (A) Q-Q plot of the −log10 p-values from a mixed linear model analysis for association of genome-wide SNPs with Herlitz type junctional epidermolysis bullosa (HJEB) in Black Headed Mutton (BHM) sheep. (B) Manhattan-plot of the −log10 p-values for the genome-wide association analysis of Herlitz type junctional epidermolysis bullosa (HJEB) in Black Headed Mutton (BHM) sheep. The peaks of the −log10 p-values are only on OAR12. (DOC) [file pone.0018943.s001.doc]
