# Supplementary material for: A Frameshift Mutation within LAMC2 Is Responsible for Herlitz Type Junctional Epidermolysis Bullosa (HJEB) in Black Headed Mutton Sheep
Source: PLoS One. 2011 May 4;6(5):e18943. doi: 10.1371/journal.pone.0018943 (PMC3087721; doi:10.1371/journal.pone.0018943)

**Figure S2.** Linkage disequilibrium (LD) of six non-conservative SNPs within *LAMC2.*The r^2^ values are shown for each SNP pair. The red square between the markers c.3001G>A and c.3455G>A indicates complete linkage.


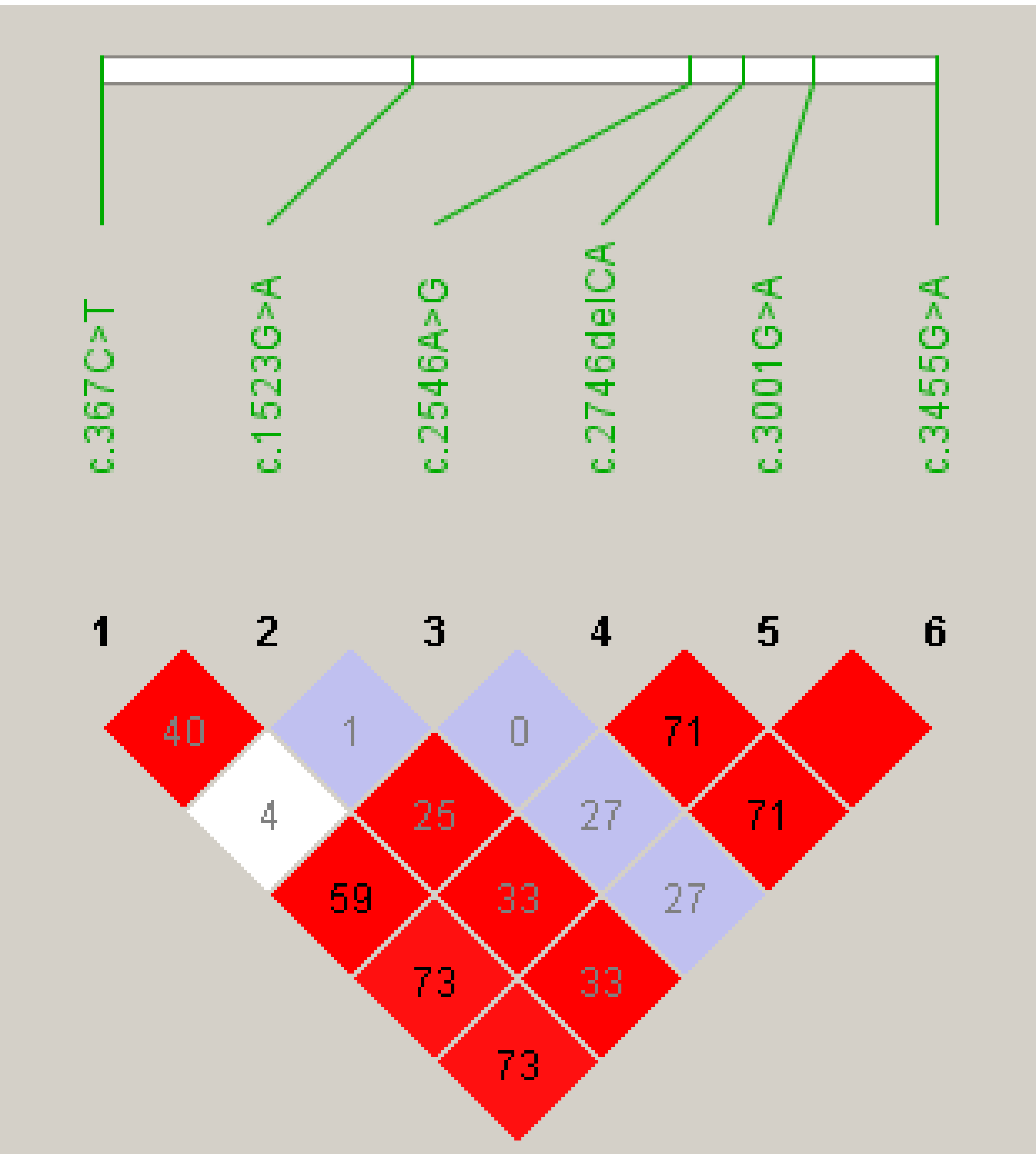

Supplement: Figure S2 — Linkage disequilibrium (LD) of six non-conservative SNPs within LAMC2 . The r2 values are shown for each SNP pair. The red square between the markers c.3001G>A and c.3455G>A indicates complete linkage. (DOC) [file pone.0018943.s002.doc]
