# Supplementary material for: A Frameshift Mutation within LAMC2 Is Responsible for Herlitz Type Junctional Epidermolysis Bullosa (HJEB) in Black Headed Mutton Sheep
Source: PLoS One. 2011 May 4;6(5):e18943. doi: 10.1371/journal.pone.0018943 (PMC3087721; doi:10.1371/journal.pone.0018943)

**Figure S3.** cDNA sequence of an HJEB-unaffected lamb (A) in comparison with the cDNA sequence of an HJEB-affected lamb (B, C). The cDNA sequence of an HJEB-affected lamb is spanning from exon 17 to exon 19 (B) and the cDNA sequence of the same affected lamb is spanning from exon 17 to intron 18 (C). The vertical line marks the end of exon 18. In the affected individual (B/C), at the end of exon 18 two bases (CA) are missing and alternative splicing results in two transcripts with one of them including 51 bp of intron 18 (B). The presence of intronic DNA within the second transcript can be clearly shown by generating sequences starting from exon 17 and ending in intron 18 (C). The boundary between exon 17 and exon 18 of all products was smooth and used to verify that only cDNA was sequenced.


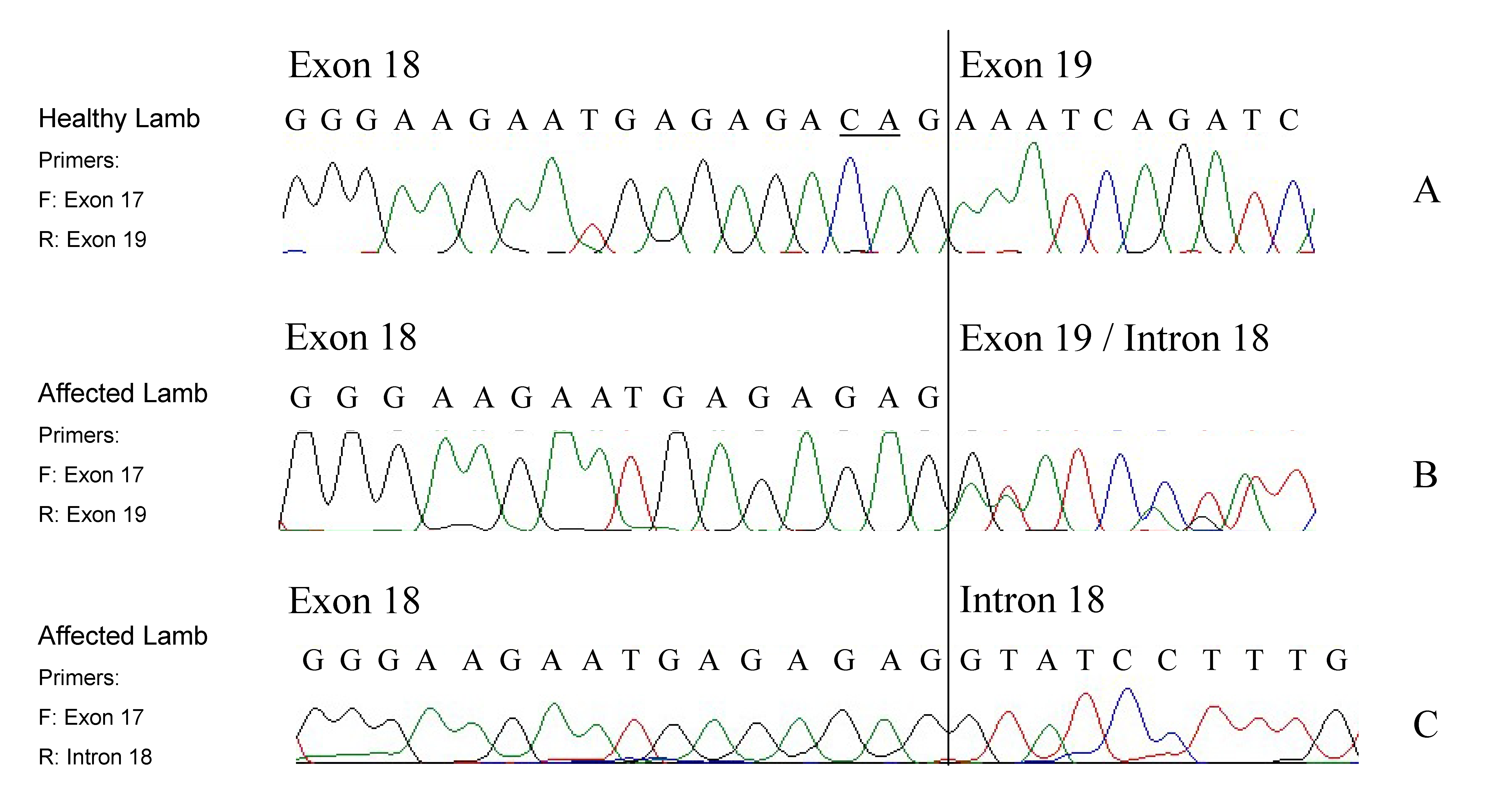

Supplement: Figure S3 — cDNA sequence of an HJEB-unaffected lamb (A) in comparison with the cDNA sequence of an HJEB-affected lamb (B, C). The cDNA sequence of an HJEB-affected lamb is spanning from exon 17 to exon 19 (B) and the cDNA sequence of the same affected lamb is spanning from exon 17 to intron 18 (C). The vertical line marks the end of exon 18. In the affected individual (B/C), at the end of exon 18 two bases (CA) are missing and alternative splicing results in two transcripts with one of them including 51 bp of intron 18 (B). The presence of intronic DNA within the second transcript can be clearly shown by generating sequences starting from exon 17 and ending in intron 18 (C). The boundary between exon 17 and exon 18 of all products was smooth and used to verify that only cDNA was sequenced. (DOC) [file pone.0018943.s003.doc]
