# Supplementary material for: A Frameshift Mutation within LAMC2 Is Responsible for Herlitz Type Junctional Epidermolysis Bullosa (HJEB) in Black Headed Mutton Sheep
Source: PLoS One. 2011 May 4;6(5):e18943. doi: 10.1371/journal.pone.0018943 (PMC3087721; doi:10.1371/journal.pone.0018943)
Supplement: Figure S4 — Cryptic intronic splice site in an HJEB-affected lamb. Section of the cDNA sequence of an HJEB-affected lamb showing the sequence of intron 18, which is spliced at the intronic base pair 51 to exon 19. The A>G mutation opening a cryptic splice site and therefore terminating this intronic insertion into the cDNA sequence is indicated. (DOC) [file pone.0018943.s004.doc]

**Figure S4.** Cryptic intronic splice site in an HJEB-affected lamb. Section of the cDNA sequence of a HJEB-affected lamb showing the sequence of intron 18, which is spliced at the intronic base pair 51 to exon 19. The A>G mutation opening a cryptic splice site and therefore terminating this intronic insertion into the cDNA sequence is indicated.

**
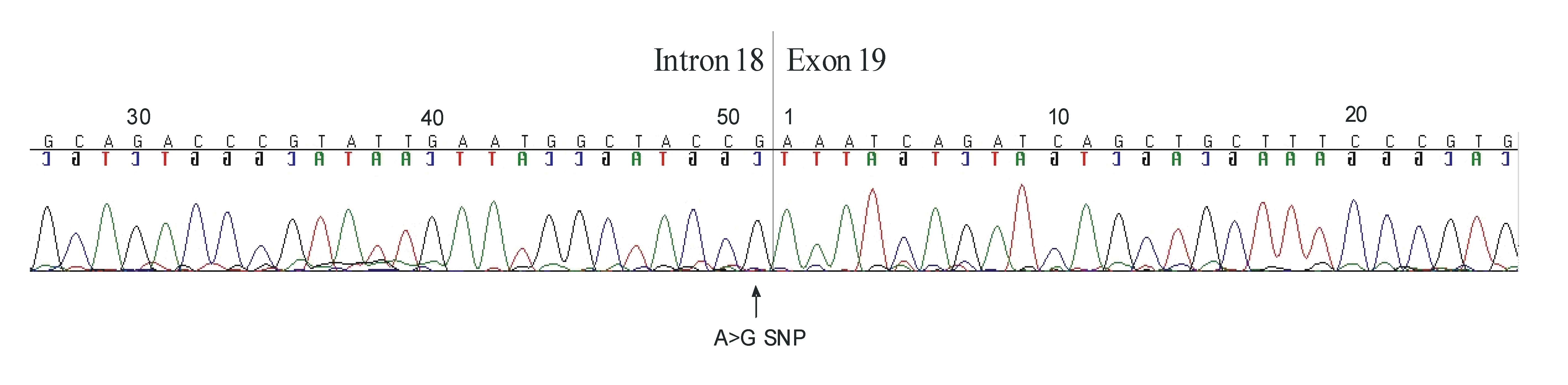
**
