# Supplementary material for: A Frameshift Mutation within LAMC2 Is Responsible for Herlitz Type Junctional Epidermolysis Bullosa (HJEB) in Black Headed Mutton Sheep
Source: PLoS One. 2011 May 4;6(5):e18943. doi: 10.1371/journal.pone.0018943 (PMC3087721; doi:10.1371/journal.pone.0018943)
Supplement: Figure S5 — Comparison of amino acid sequences among an unaffected and an HJEB-affected animal. Sequence labelled with 1 is from an unaffected lamb and sequences labelled 2 and 3 are from one HJEB-affected lamb. Sequence 2 demonstrates the transcript with correct and sequence 3 demonstrates the transcript with alternative splicing. Both variants (2 and 3) were present in every HJEB-affected lamb and both sequences lead to a premature stop codon and thus to a shortened, non-functional protein. (DOC) [file pone.0018943.s005.doc]

**Figure S5.** Comparison of amino acid sequences among an unaffected and an HJEB-affected animal. Sequence labelled with 1 is from an unaffected lamb and sequences labelled 2 and 3 are from one HJEB-affected lamb. Sequence 2 demonstrates the transcript with correct and sequence 3 demonstrates the transcript with alternative splicing. Both variants (2 and 3) were present in every HJEB-affected lamb and both sequences lead to a premature stop codon and thus to a shortened, non-functional protein.

1- MPALWLSGCLCLSLLLPAARANSRTPVCDCNGKSRQCIFDQELHRLTGNGFRCLHCSDNT

2- MPALWLSGCLCLSLLLPAARANSRTPVCDCNGKSRQCIFDQELHRLTGNGFRCLHCSDNT

3- MPALWLSGCLCLSLLLPAARANSRTPVCDCNGKSRQCIFDQELHRLTGNGFRCLHCSDNT

************************************************************

1- GGIHCERCREGFYRHRERDRCLPCNCNSKGSLSPRCDNTGRCSCKPGVTGDRCDRCLPGF

2- GGIHCERCREGFYRHRERDRCLPCNCNSKGSLSPRCDNTGRCSCKPGVTGDRCDRCLPGF

3- GGIHCERCREGFYRHRERDRCLPCNCNSKGSLSPRCDNTGRCSCKPGVTGDRCDRCLPGF

************************************************************

1- HTLTDAGCTQDRKLLDSKCDCDPAGIVGPCDTGRCVCKPAVTGERCDRCRPGYYHLDGRN

2- HTLTDAGCTQDRKLLDSKCDCDPAGIVGPCDTGRCVCKPAVTGERCDRCRPGYYHLDGRN

3- HTLTDAGCTQDRKLLDSKCDCDPAGIVGPCDTGRCVCKPAVTGERCDRCRPGYYHLDGRN

************************************************************

1- PEGCTQCFCYGHSASCQSSGDYSVHKILSTFHQDVDGWKAVQRNGFPAKLQWSQRHQDVF

2- PEGCTQCFCYGHSASCQSSGDYSVHKILSTFHQDVDGWKAVQRNGFPAKLQWSQRHQDVF

3- PEGCTQCFCYGHSASCQSSGDYSVHKILSTFHQDVDGWKAVQRNGFPAKLQWSQRHQDVF

************************************************************

1- SSARRSDPVYFVAPAKFLGNQQVSYGQSLSFDYRVDRGGRHPSAHDVILEGAGLQITAPL

2- SSARRSDPVYFVAPAKFLGNQQVSYGQSLSFDYRVDRGGRHPSAHDVILEGAGLQITAPL

3- SSARRSDPVYFVAPAKFLGNQQVSYGQSLSFDYRVDRGGRHPSAHDVILEGAGLQITAPL

************************************************************

1- MPRDKTLPCGITKTYTFRLNEHPSSNWRPQLSYFEYRRLLRNLTALRIRATYGEYSTGYL

2- MPRDKTLPCGITKTYTFRLNEHPSSNWRPQLSYFEYRRLLRNLTALRIRATYGEYSTGYL

3- MPRDKTLPCGITKTYTFRLNEHPSSNWRPQLSYFEYRRLLRNLTALRIRATYGEYSTGYL

************************************************************

1- DNVTLISARPISGTPAPWVEQCVCPVGYKGQFCQECASGYKRDSARLGPFGTCIPCNCQG

2- DNVTLISARPISGTPAPWVEQCVCPVGYKGQFCQECASGYKRDSARLGPFGTCIPCNCQG

3- DNVTLISARPISGTPAPWVEQCVCPVGYKGQFCQECASGYKRDSARLGPFGTCIPCNCQG

************************************************************

1- GGACDPDTGDCYSGDENPDIECADCPIGFYNDPHDPRSCKPCPCRNGFSCSVMPETEEVV

2- GGACDPDTGDCYSGDENPDIECADCPIGFYNDPHDPRSCKPCPCRNGFSCSVMPETEEVV

3- GGACDPDTGDCYSGDENPDIECADCPIGFYNDPHDPRSCKPCPCRNGFSCSVMPETEEVV

************************************************************

1- CNNCPHGVTGARCELCADGYFGDPFGERGPVRPCQPCQCSNNVDPNAPGNCDRLTGRCLK

2- CNNCPHGVTGARCELCADGYFGDPFGERGPVRPCQPCQCSNNVDPNAPGNCDRLTGRCLK

3- CNNCPHGVTGARCELCADGYFGDPFGERGPVRPCQPCQCSNNVDPNAPGNCDRLTGRCLK

************************************************************

1- CLYNTTGAHCDQCKAGYYGDPLAPNPADKCRACNCNPMGSGPVECRSDGSCVCKPGFDGL

2- CLYNTTGAHCDQCKAGYYGDPLAPNPADKCRACNCNPMGSGPVECRSDGSCVCKPGFDGL

3- CLYNTTGAHCDQCKAGYYGDPLAPNPADKCRACNCNPMGSGPVECRSDGSCVCKPGFDGL

************************************************************

1- HCDHAALINCPACYNQVKTQMDQFMQQLESLETLLSKAQAGGGAVPDAELEGRMQQAEQA

2- HCDHAALINCPACYNQVKTQMDQFMQQLESLETLLSKAQAGGGAVPDAELEGRMQQAEQA

3- HCDHAALINCPACYNQVKTQMDQFMQQLESLETLLSKAQAGGGAVPDAELEGRMQQAEQA

************************************************************

1- LQDLLREAQISEGAIRSLNLQLAKARSQENSYRTRLDDLKMTVERLRTLGSQHQDRVQDT

2- LQDLLREAQISEGAIRSLNLQLAKARSQENSYRTRLDDLKMTVERLRTLGSQHQDRVQDT

3- LQDLLREAQISEGAIRSLNLQLAKARSQENSYRTRLDDLKMTVERLRTLGSQHQDRVQDT

************************************************************

1- RRLITQMRLSLEESEAALRNTNIPPSEHYTGPNGFKSLAQEATRLADSHVESANNMEQLV

2- RRLITQMRLSLEESEAALRNTNIPPSEHYTGPNGFKSLAQEATRLADSHVESANNMEQLV

3- RRLITQMRLSLEESEAALRNTNIPPSEHYTGPNGFKSLAQEATRLADSHVESANNMEQLV

************************************************************

1- RETEDYSKQALTLARKAATEGGSSGSLRGSVVQELVGKLEKTKSLAQQLSREATQIDTEA

2- RETEDYSKQALTLARKAATEGGSSGSLRGSVVQELVGKLEKTKSLAQQLSREATQIDTEA

3- RETEDYSKQALTLARKAATEGGSSGSLRGSVVQELVGKLEKTKSLAQQLSREATQIDTEA

************************************************************

1- DTSYQHSLHLLSSATQLQGVSDQSFQVEAKKIRQKADSLSSLVTKRMDEFKRVQSSLGNW

2- DTSYQHSLHLLSSATQLQGVSDQSFQVEAKKIRQKADSLSSLVTKRMDEFKRVQSSLGNW

3- DTSYQHSLHLLSSATQLQGVSDQSFQVEAKKIRQKADSLSSLVTKRMDEFKRVQSSLGNW

************************************************************

1- EEETQKLLQDGKNERQKSDQLLSRANLAKSRAQEALSMGNATFYEVENILKNLREFDLQV

2- EEETQKLLQDGKNEREIRSAAFPCQPC*

3- EEETQKLLQDGKNERGILCYSVPSADPY*

***************

1- EDRKAEAEEAMKRLSYISQRVADASDKTRRAETALGGAATDAQRAKTAAGEALNIAGKIE

2-

3-

1- QEIGSLNLEANVTADGALAMEKGLATLKSEMRKVEGELARKEREFDVDMDAVQTVIAEAQ

2-

3-

1- RADSRAENAGVTIQDTLDTLDSILHLIDQPGSVDEEGLISLEQKLFRAKTQINSQLRPLM

2-

3-

1- SELEERVRWQWGHLRSLETSIDGILADVKNLETIRDSLPPGCYNTQALEQH*

2-

3-
