# Supplementary material for: A Frameshift Mutation within LAMC2 Is Responsible for Herlitz Type Junctional Epidermolysis Bullosa (HJEB) in Black Headed Mutton Sheep
Source: PLoS One. 2011 May 4;6(5):e18943. doi: 10.1371/journal.pone.0018943 (PMC3087721; doi:10.1371/journal.pone.0018943)

**Figure S6.** Pedigree of the HJEB-affected Black Headed Mutton sheep. Sheep from the three farms are marked by grey boxes in contrast to the progeny born in the mating experiment. Animals marked by an asterisk were genotyped on the ovine Illumina 50K beadchip.


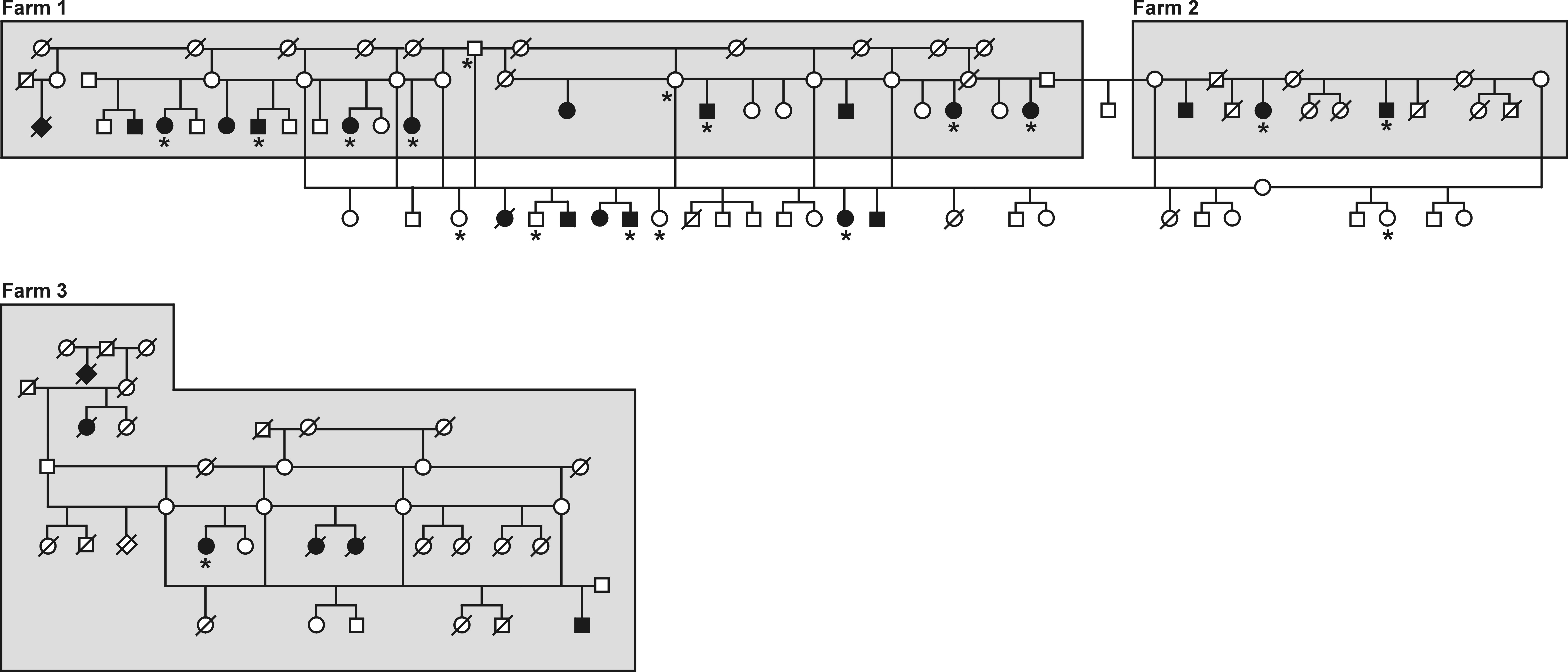

Supplement: Figure S6 — Pedigree of the HJEB-affected Black Headed Mutton sheep. Sheep from the three farms are marked by grey boxes in contrast to the progeny born in the mating experiment. Animals marked by an asterisk were genotyped on the ovine Illumina 50 K beadchip. (DOC) [file pone.0018943.s006.doc]
