# Supplementary material for: A Frameshift Mutation within LAMC2 Is Responsible for Herlitz Type Junctional Epidermolysis Bullosa (HJEB) in Black Headed Mutton Sheep
Source: PLoS One. 2011 May 4;6(5):e18943. doi: 10.1371/journal.pone.0018943 (PMC3087721; doi:10.1371/journal.pone.0018943)
Supplement: Table S1 — Polymorphisms identified within ovine LAMA3 and LAMB3 used for linkage analysis and their location, polymorphism information content (PIC) and heterozygosity (HET). (DOC) [file pone.0018943.s007.doc]

**Table S1.** Polymorphisms identified within ovine *LAMA3* and *LAMB3* used for linkage analysis and their location, polymorphism information content (PIC) and heterozygosity (HET).

| Gene | Nomenclature | Location | PIC | HET |
| --- | --- | --- | --- | --- |
| *LAMA3* | FM872294:g.226T>G | intron 61 | 0.33 | 0.45 |
|  | FM872294:g.378C>T | intron 61 | 0.33 | 0.45 |
|  | FM872294:g.415G>A | intron 61 | 0.33 | 0.45 |
|  | FM872294:g.511A>G | intron 61 | 0.33 | 0.45 |
|  | FM872294:g.549C>A | intron 61 | 0.33 | 0.45 |
|  | FM872294:g.601T>C | intron 61 | 0.33 | 0.45 |
|  | FM872294:g.609C>T | intron 61 | 0.33 | 0.46 |
|  | FM872294:g.533(TA)4 | intron 61 | 0.33 | 0.46 |
| *LAMB3* | FN429931:g.202C>T | 5’UTR | 0.42 | 0.41 |
|  | FN429931:g.816G>T | intron 1 | 0.28 | 0.43 |
|  | FN429932:g.64G>A | intron 2 | 0.11 | 0.12 |
|  | FM872309:g.82G>A | intron 3 | 0.05 | 0.03 |
|  | FM872309:g.97C>T | intron 3 | 0.05 | 0.03 |
|  | FM872309:g.260A>G | intron 3 | 0.05 | 0.03 |
|  | FM872309:g.361C>T | intron 3 | 0.36 | 0.41 |
|  | FM872309:g.436T>C | intron 3 | 0.05 | 0.03 |
|  | FM872309:g.615C>T | intron 3 | 0.07 | 0.03 |
|  | FN429933:g.128A>G | exon 8 | 0.14 | 0.17 |
|  | FN429933:g.203C>A | intron 8 | 0.14 | 0.17 |
|  | FN429933:g.249G>C | intron 8 | 0.14 | 0.17 |
|  | FN429933:g.256A>G | intron 8 | 0.14 | 0.17 |
|  | FN429933:g.292A>G | intron 8 | 0.14 | 0.17 |
|  | FN429933:g.403T>A | intron 8 | 0.14 | 0.17 |
