# Supplementary material for: A Frameshift Mutation within LAMC2 Is Responsible for Herlitz Type Junctional Epidermolysis Bullosa (HJEB) in Black Headed Mutton Sheep
Source: PLoS One. 2011 May 4;6(5):e18943. doi: 10.1371/journal.pone.0018943 (PMC3087721; doi:10.1371/journal.pone.0018943)
Supplement: Table S2 — Mutations within LAMC2 causing Herlitz and Non-Herlitz JEB in human and horse. (DOC) [file pone.0018943.s008.doc]

**Table S2.** Mutations within *LAMC2* causing Herlitz and Non-Herlitz JEB in human and horse.

| Species | Mutation | Location | Type |
| --- | --- | --- | --- |
| Human | p.Q46X | Exon 2 | Non-Herlitz |
| Human | p.R95X | Exon 3 | Herlitz |
| Human | c. 405-1G>A | Intron 3 | Non-Herlitz |
| Human | c.404+3del4 | Intron 3 | Herlitz |
| Human | c.525delTC | Exon 5 | Non-Herlitz |
| Human | p.Q186X | Exon 5 | Herlitz |
| Human | p.R245X | Exon 6 | Non-Herlitz |
| Human | c.764-10T>G | Intron 6 | Herlitz |
| Human | c.953G>T | Exon 7 | Herlitz |
| Human | c.1037delT | Exon 8 | Herlitz |
| Human | p.R349X | Exon 8 | Herlitz |
| Human | p.Y355X | Exon 8 | Herlitz |
| Human | c.1067-1G>A | Intron 8 | Herlitz |
| Human | p.C553X | Exon 11 | Herlitz |
| Human | c.2137del7 | Exon 14 | Herlitz |
| Human | c.2219del20>G | Exon 14 | Non-Herlitz |
| Human | p.K822X | Exon 17 | Herlitz |
| Human | p.Q864X | Exon 17 | Herlitz |
| Human | p.Q896X | Exon 18 | Herlitz |
| Human | c.2869+1G>A | Intron 19 | Herlitz |
| Human | c.3235delA | Exon 22 | Herlitz |
| Human | c.3394insA | Exon 23 | Non-Herlitz |
| Horse | AF533668:c.1368insC | Exon 10 | Herlitz |
