# Supplementary material for: A Frameshift Mutation within LAMC2 Is Responsible for Herlitz Type Junctional Epidermolysis Bullosa (HJEB) in Black Headed Mutton Sheep
Source: PLoS One. 2011 May 4;6(5):e18943. doi: 10.1371/journal.pone.0018943 (PMC3087721; doi:10.1371/journal.pone.0018943)
Supplement: Table S3 — Primer sequences with their product sizes (P) and annealing temperatures (AT) used for amplification of sequences within LAMA3 , LAMB3 , and LAMC2 as well as the targeted regions within these genes. (DOC) [file pone.0018943.s009.doc]

**Table S3.** Primer sequences with their product sizes (P) and annealing temperatures (AT) used for amplification of sequences within *LAMA3*, *LAMB3*, and *LAMC2* as well as the targeted regions within these genes.

| Gene | Target | Template | Primer F (5’->3’) | Primer R (5’->3’) | P | AT |
| --- | --- | --- | --- | --- | --- | --- |
| LAMA3 | exon 61-62 | gDNA | TGTAGAAGATGGCCGACTCA | CCCAGATAGTATGTGCTGAAATC | 870 | 59 |
|  | intron 61 | gDNA | CAGACAAACAAATGCAAACATCT | TTTTGCCTTCCTTCAGATTCA | 133 | 59 |
| LAMB3 | intron 3 | gDNA | TGACTGATTGGATCTTCTTGG | ATGCTGTGACTGGGAGAGAG | 697 | 58 |
|  | 5’ UTR, exon 1 | gDNA | GGAATCCTGGTCTCCTGTGA | GCGGTTCTCTCTCCAGATCA | 323 | 60 |
|  | 5’ UTR, exon 1 | gDNA | GGGAGTCTCCAGAGGTGA | CTCGAAGGGTCCTAGAGGC | 787 | 60 |
|  | intron 2 | gDNA | ATGGGTGGGAGGACAGTCTA | AAAAGCAGAAACTGATCCTGAAA | 770 | 59 |
|  | exon 8, intron 8 | gDNA | GAGGGTCAACTTCACCAAGC | GCCTTAAGTTCCCTGCGATT | 458 | 60 |
| LAMC2 | exon 1-6 | cDNA | ATGCCTGCGCTCTGGCTC | ACCCATTTCTCTGGACAGC | 676 | 58 |
|  | exon 5-9 | cDNA | TCACCTGGATGGGAGAAAC | TTTGTAGCCAGAAGCACATTC | 682 | 58 |
|  | exon 8-10 | cDNA | GCTGAGTTACTTTGAGTATCG | GCAGTTATTGCACACCACCT | 463 | 60 |
|  | exon 9-12 | cDNA | GTGCTTCTGGCTACAAAAGA | CTTCACTTGATTATAGCAAGC | 668 | 58 |
|  | exon 11-13 | cDNA | GCTACTTTGGGGACCCCTT | AGCCTGCTGCATCCTGCC | 476 | 60 |
|  | exon 12-14 | cDNA | GTGATGGCAGCTGTGTTTG | CGGGTCCGGTAGCTATTC | 325 | 58 |
|  | exon 13-18 | cDNA | ACTGCTTTCGAAGGCTCAG | CATCCATTCGCTTAGTCACC | 767 | 56 |
|  | exon 17-19 | cDNA | AAAATTGGAGAAAACCAAGTC | ACTTCATAAAAAGTGGCATTGC | 387 | 60 |
|  | exon 17 - intron 18 | cDNA | TCAGCTTCAGGGAGTCAGTG | AAGGAACGGAGTAACAAAGGATAC | 208* | 60 |
|  | intron 18 – exon 20 | cDNA | ACTCCGTTCCTTCAGCAGAC | TGGTCTTGTCACTGGCATCT | 256* | 60 |
|  | exon 18-19 | cDNA | CCTTCCAGGTAGAAGCAAAGAA | CAACTTCATAAAAAGTGGCATTG | 249 | 60 |
|  | exon 15-20 | cDNA | GCCAAATGGCTTTAAAAGTCT | CTGGCATCTGCAACCCTCT | 702 | 58 |
|  | exon 19-23 | cDNA | GGGCAATGCCACTTTTTATG | CTTCACATCAGCCAGAATGC | 694 | 59 |
|  | exon 23 – 3`UTR | gDNA | CGAGCCAAGACCCAGATC | TTACTGACCACAGCATGACTTC | 660 | 58 |
|  | 3`UTR | gDNA | TGATGCTCTTTGCTTCCTGA | GTGAGAACAAAACCCACAGG | 688 | 58 |
|  | 3`UTR | gDNA | TTTCCCCTGATGATGCTAGG | ACAGAGGTGAGGCAGTACCA | 722 | 58 |
|  | exon 3 | gDNA | CTCGATGTGACAACACTGG | CGGTCTTGGGTGCACCC | 115 | 58 |

*When sequencing alternative splice variant

**Table S3.** Continued.

| LAMC2 | intron 4-5 | gDNA | AAAGTAGAAATGATCAGTCACC | AATAGGAAATGCAGGGAGTTG | 501 | 60 |
| --- | --- | --- | --- | --- | --- | --- |
|  | exon 11 | gDNA | CTACTTTGGGGACCCCTTTG | CGACACTTGTCCGCTGGG | 216 | 58 |
|  | exon 13 | gDNA | AGATGGATCAGTTTATGCA | CTGAAATCTGGGCTTCTCT | 156 | 53 |
|  | exon 13-14 | gDNA | ACTGCTTTCGAAGGCTCAG | CGGGTCCGGTAGCTATTC | 479 | 58 |
|  | exon 16-17 | gDNA | GCCAATAACATGGAGCAACT | GGAAGGACTGATCACTGAC | 804 | 58 |
|  | exon 18, intron 18 | gDNA | GAGTTCAAGCGTGTGCAAAG | GGCAGCAATACCATGACCTT | 182 | 60 |
|  | exon 20 | gDNA | GCAGGTTGAAGACAGAAAAG | GATCTTGCCGGCGATATTC | 184 | 58 |
|  | exon 22 | gDNA | GTGATTGCAGAAGCCCAGA | CTATTAGATGTAGGATGCTAT | 100 | 58 |

*When sequencing alternative splice variant
